# Concerted Efforts Are Needed to Control and Mitigate Antibiotic Pollution in Coastal Waters of China

**DOI:** 10.3390/antibiotics9020088

**Published:** 2020-02-16

**Authors:** Huaijun Xie, Jing Du, Jingwen Chen

**Affiliations:** 1 Key Laboratory of Industrial Ecology and Environmental Engineering (MOE), School of Environmental Science and Technology, Dalian University of Technology, Dalian 116024, China; huaijunxie@dlut.edu.cn; 2 Dalian Key Laboratory of Conservation Biology for Endangered Marine Mammals, Liaoning Ocean and Fisheries Science Research Institute, Dalian 116023, China; dujingdl@mail.dlut.edu.cn

**Keywords:** antibiotic pollution, coastal water, perspective

## Abstract

Antibiotics have been applied for decades and antibiotic pollution is of great concern due to the risk for promoting resistant genes. Human activities such as mariculture and land-based discharge can lead to the antibiotic pollution in coastal area and it is of importance to assess the pollution and risks of antibiotics in this area. In this mini-review, the pollution status of antibiotics in Chinese coastal waters is summarized and some perspectives are put forward for future efforts to mitigate the pollution.

## 1. Introduction

The application of antibiotics worldwide has been increasing since 1940s. Particularly, China is estimated to consume the most antibiotics in the world with the consumption amounting to more than 160,000 tons in 2013. These antibiotics are used to cure or prevent diseases for human or applied in stock farming and aquaculture [1]. It is known that the applied antibiotics could not be absorbed or metabolized entirely in organisms and about 30%–90% of them would be released to the environment. Although some antibiotics undergo degradation [2] (e.g., photodegradation [3,4,5,6] and hydrolysis [7]) in the environment, their environmental levels can still increase if their general emission rates are higher than the degradation rates.

About two-thirds of global rivers carry organic micropollutants including antibiotics, pesticides, and industrial chemicals to estuarine and coastal areas which are dynamic ecosystems hosting some of the highest biodiversity and biological production in the world. Thus, it is of importance to assess the pollution and risks of antibiotics in estuarine and coastal areas [8]. In this mini-review, the pollution of antibiotics in Chinese coastal waters is summarized so as to get an aerial view on the pollution status and to suggest future efforts to mitigate the pollution. 

## 2. Occurrence and Distribution of Antibiotics in Coastal Waters of China

**Figure 1:**
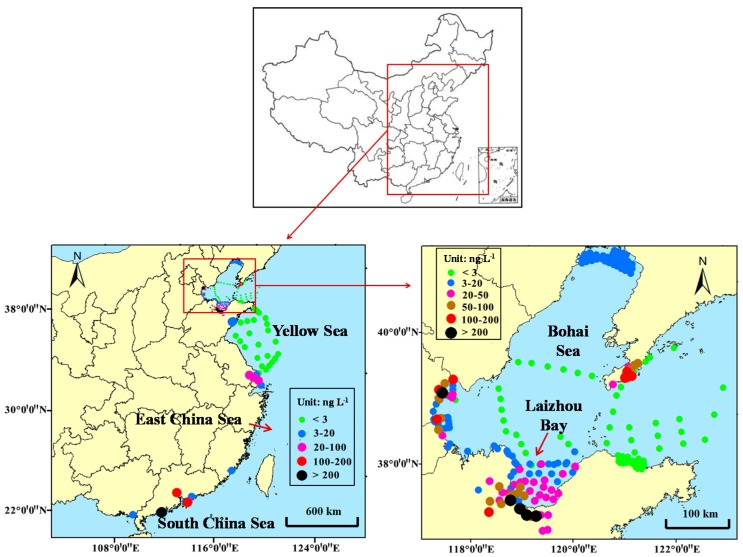
Levels of antibiotics in coastal water around China [9,10,11,12,13,14,15,16,17,18,19,20,21,22].

**Figure 2:**
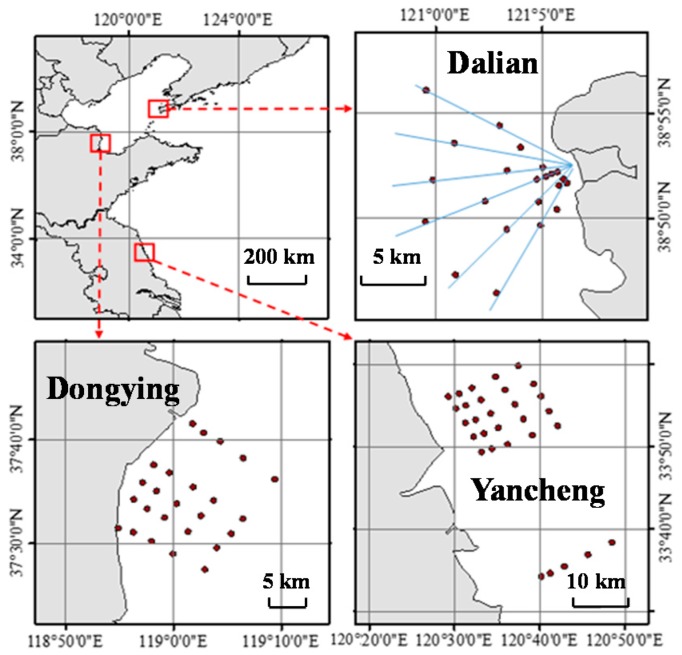
Sampling sites of coastal waters for detection of antibiotics.

Since 2007 when Xu et al. reported determination of selected antibiotics in the Victoria harbor and the Pearl river, South China, there have been some investigations and reports on antibiotic levels in rivers and coastal waters in China [9]. These studies mainly focused on coastal waters in four seas; the Bohai Sea, the Yellow Sea, the East China Sea, and the South China Sea (Figure 1). As the Bohai Sea is an interior sea of China and has poorer water exchange ability, most previous studies focused on the Bohai Sea. Based on results from previous studies, levels of antibiotics in estuarine and coastal waters in China are summarized and indicated in Figure 1 and Table 1 [9,10,11,12,13,14,15,16,17,18,19,20,21,22]. More than 36 antibiotics have been detected in the coastal waters and their concentrations generally range from several ng L^−1^ to dozens of ng L^−1^. However, the levels could be even up to μg L^−1^ in some heavily polluted areas. For example, norfloxacin was once determined to be 6.8 μg L^−1^ in the Bohai Bay. Sulfonamides, macrolides, and fluoroquinolones were found to have higher detection rates and levels than others, especially for sulfamethoxazole, trimethoprim, norfloxacin, enrofloxacin, and erythromycin [21]. Our previous studies examined antibiotics in three Chinese coastal areas (Figure 2) with concentration scales of 33.2–136.9 ng L^−1^(Dalian), 62.3–316.4 ng L^−1^(Dongying), and 1.9–94.9 ng L^−1^(Yancheng), respectively (data not published).

The pollution levels in the Bohai Sea are generally higher than those in the other seas, which mainly results from the poorer water exchange of the Bohai Sea. A negative relationship was found between the levels of antibiotics and distances from coastline to the sampling sites. This can be explained by the effects of dilution, degradation, and adsorption by sediments and suspended particles [23].

Although continuous monitoring data on antibiotics in Chinese coastal waters are very limited, it can still be inferred that antibiotic levels generally have an increasing trend in the coastal waters. For example, the antibiotics levels in the Victoria harbor in the South China Sea were mainly below the limit of quantification and the maximum detected level was 30.6 ng L^−1^ in 2007. While in 2015, 21 antibiotics were detected in the Hailing Bay of the South China Sea with levels up to 15,163 ng L^−1^ [9]. A similar trend can be also observed in the coastal waters around Dalian by comparing the levels determined in 2013 [24] (2.11–9.23 ng L^−1^) [13] and in 2016 (33.2–136.9 ng·L^−1^, unpublished data).

## 3. Potential Sources of Antibiotic Pollution in Coastal Waters 

Riverine transport, discharge from land-based sources and mariculture activities are the main sources of antibiotics pollution in coastal waters in China. It was estimated that humans consumed about 48% of the 160,000 tons of antibiotics and the rest were shared by animals in 2013. Zhang et al. [1] determined antibiotics in the Laizhou Bay and 10 ambient rivers, and found antibiotics levels in rivers were much higher than those in the seawaters. Zou et al. [18] investigated antibiotics levels in rivers and mariculture ponds adjacent to the Bohai Bay and got a similar conclusion. Sulfonamides and tetracyclines were shown to be predominant antibiotics in the fish farming ponds in China’s southeast coast [21]. In addition, streptomycin, neomycin, penicillin, and rifampin were proved to be effective for the development and survival of giant clams and therefore they were widely used in the mariculture [25,26]. These results indicate that antibiotics in rivers and ponds could continually flow into coastal waters and lead to the pollution. The mariculture mainly include in land- or sea-based enclosures, such as cages, ponds, or raceways. It seems that the relatively closed ones may release more antibiotics to the seawaters.

## 4. Risks of Antibiotics in Marine Environment

Although levels for most antibiotics in the coastal waters mentioned above were proved to have no acute toxicity for marine organisms [16,18,24], the ecological risks of antibiotics in the coastal waters should not be ignored, as many studies indicated that antibiotics in coastal waters may promote the selection of antibiotic resistances [27,28,29]. For example, Niu et al. [30] found that the abundance of resistance genes has a positive relationship with the levels of antibiotics in the Bohai Bay. The antibiotics in the coastal waters can also be adsorbed and stored by sediments, promoting the occurrence of resistance genes among abundant microbes in the sediments. The resistance genes in the marine environment have the potential to enter the biosphere [19,31]. It has been reported that fishmeal is a major reservoir for resistance genes. Table 2 shows relative information on the antimicrobial resistance genes detected in marine environment of China [32]. Besides promoting resistance, some antibiotics of high levels may also pose direct toxicity to the marine organisms. Chen et al. [24] pointed out that erythromycin-H_2_O, norfloxacin, and oxytetracycline in seawaters of the Hailing Bay could pose high risks to marine sensitive species. 

Actually, the antibiotics do not exist in the aquatic environment separately. Previous studies showed that the combined antibiotics can exhibit synergistic effects and lead to joint ecotoxicity. For example, synergistic effects were observed when combinations of amoxicillin, erythromycin, levofloxacin, norfloxacin, and tetracycline were tested on cyanobacterium and green algae [36,37]. It is indicated that specific combinations of antibiotics at the present environmental levels can pose a potential ecological risk for aquatic ecosystems [37,38,39].

Conventionally, only hydrophobic pollutants were considered to be bioaccumulative. However, it was found that some antibiotics that are not very hydrophobic, including sulfonamides, fluoroquinolones, and macrolides, can also be bio-concentrated in marine animals and sulfonamides can even be biomagnified in the food web of the Laizhou Bay [40]. 

## 5. Perspective

In recent years, the Chinese government has promulgated a series of policies to improve the environmental quality and to build the “Beautiful China”, including management of the antibiotic pollution in aquatic environments. For example, the “Action Plan for Prevention and Control of Water Pollution” put forward by the state council in 2015 stipulates “To strengthen management of aquaculture inputs, standardize and limit the use of chemical medicine such as antibiotics in accordance with the law”. In 2019, the “No 1 Central Document”, the first policy statement released by central authorities each year, holds that “To control and reduce the scale of inland and coastal aquaculture”, which can also reduce the antibiotics consumption and emission. Therefore, it is expected that the antibiotic levels in coastal waters will decrease in the future. 

To evaluate the performance of the national policies and to provide sound technologies to control and mitigate the pollution of antibiotics in the coastal waters, the following measures can be implemented. 

(1) Systematical surveys on spatial and temporal variation of antibiotics in coastal waters should be organized. There are currently many data gaps on levels of antibiotics in the coastal waters with regard to the spatial and temporal scales. The previous studies are far from enough to reflect the pollution characteristics of antibiotics in the coastal waters. 

Due to the broad areas of coastal waters relative to inland rivers, collecting and transporting seawater samples for target pollutants analysis is very laborious, costly, and time-consuming. Development and application of passive sampling or other in situ novel active sampling technology (e.g., osmotic sampler [41]) becomes important. Passive sampling technique, e.g., the diffusive gradients in thin-films (DGT) technique [42], can provide a time-weighted average concentration of pollutants. Chen et al. developed DGT techniques to monitor antibiotics in freshwaters. Xie et al. [43,44] further developed DGT technology to monitor antibiotics and endocrine disrupting chemicals in seawaters [45,46].

As coastal waters are sinks for many organic and inorganic pollutants, our understanding on diversity of organic micropollutants in the coastal waters can be very limited. To protect ecosystems of coastal zones, non-target analytical methods can be developed to screen other emerging organic micropollutants in the coastal waters. 

(2) Environmental behavior and ecological risks of antibiotics in the coastal waters should be investigated, so as to assess environmental capacity of the pollutants and manage the application of antibiotics. For example, some studies have indicated photodegradation in a dominant degradation pathway of some antibiotics (e.g., ciprofloxacin [4], sulfadiazine [47], and sulfapyridine [48]). It was also found that dissolved organic matter (DOM) from coastal waters impacted by mariculture exhibited higher promotion effects on photodegradation of sulfonamide antibiotics than that from offshore seawaters. Nevertheless, the effects of DOM in coastal waters influenced by different land-based sources on photodegradation of antibiotics and other micropollutants are largely unknown and need further investigations [6]. It is also important to understand the photodegradation pathways of micropollutants such as antibiotics in coastal waters, and to develop prediction models on photodegradation kinetics in coastal water bodies [49]. 

(3) More studies are necessary to investigate pollution control technology for antibiotic containing wastewaters from hospitals, pharmaceutical factories, livestock and poultry farms, and even indoor industrial type of aquaculture plants. It is reported that the conventional activated sludge methods were negative for removing antibiotics in waste waters [50]. Therefore more effective and economical methods should be developed [51]. Previous studies proved high efficiency of UV activation of hydrogen peroxide for antibiotic removal in aqueous solutions. This technique can be a potential waste water treatment method for the discharge of antibiotics [52]. Further attentions should be paid on more suitable reaction conditions and decreasing the cost of treatment. Meanwhile, it is also necessary to reduce stocking density for aquiculture ponds, and to develop greener aquiculture technologies [53].

## Figures and Tables

**Table 1:** Level range and mean level of antibiotics in coastal water around China (unit: ng L^-1^) [9,10,11,12,13,14,15,16,17,18,19,20,21,22].

Antibiotic	Level Range	Mean Level	Antibiotic	Level Range	Mean Level
**Sulfonamides**			**Tetracycline**		
Sulfadiazine	0.1–209.0	14.2	Tetracycline	1.0–122.0	23.9
Sulfacetamide	0.3–56.8	14.7	Doxycycline	0.3	0.3
Sulfadiazole	0.1–52.8	4.7	Chlortetracycline	0.6–5.0	1.8
Sulfamethoxine	0.2–41.7	8.4	Methyl cyclin	2.1–2.3	2.2
Sulfachloropyridazine	0.2–233.2	65.1	Oxytetracycline	2.5–15,163.0	578.9
Sulfamethoxazine	0.2–86.4	26.0	**Chloramphenicol**		
Sulfamonomethoxine	0.1–28.9	7.3	Chloramphenicol	0.2–0.9	0.4
Sulfadimethoxine	0.3–108.4	20.4	Thiamphenicol	0.8–85.0	24.9
Sulfamethoxazole	0.2–47.2	8.9	Florfenicol	0.5–40.0	11.6
Sulfamethoxazole	0.1–527.0	19.1	**Macrolides**		
Acetyl Sulfamethoxazole	5.9–52.8	25.6	Roxithromycin	0.1–630.0	38.4
Sulfadiazine	0.1–30.0	3.4	Azithromycin	0.1–396.0	45.2
Sulfaguanidine	0.6–3.7	1.5	Erythromycin	0.1–486.0	16.9
Sulfanilamide	0.5–7.9	2.5	Clarithromycin	0.2–32.9	3.1
Sulfaquinoxaline	0.5–7.0	1.9	**β-lactam**		
**Quinolone**			Cephalexin	10.0–182.0	43.7
Norfloxacin	2.3–6800.0	129.3	Cefradine	5.3–90.0	41.8
Enoxacin	23.4–508.0	98.8	**Others**		
Ofloxacin	0.8–5100.0	57.0	Salinomycin	1.3–36.9	9.9
Enrofloxacin	1.9–24.6	111.5	Trimethoprim	1.3–13,600.0	416.1
Ciprofloxacin	3.3–39.0	9.7			

**Table 2:** Information of antimicrobial resistance genes detected in marine environment of China.

Antimicrobial Resistance Genes	Geographic Locations	Local Factors	Reference
*cat* I, *cat* III	Jiaozhou Bay	Surface seawater	[33]
*cat* II, *cat* IV, *flo*R, *tet*B, *tet*D, *tet*E, *tet*M	Around Dalian	Maricultural environments	[34]
*sul*1, *sul*2, *tet*A, *tet*C, *tet*D, *qnr*S, *qnr*B, *qnr*A	Around Dalian	Maricultural environments	[35]
*sul*1, *sul*2, *sul*3, *sul*P, *tet*A, *tet*B, *tet*C, *tet*D, *qnr*S, *qnr*B	Around Tangshan	Maricultural environments	[35]
*sul*1, *sul*2, *sul*P, *tet*A, *tet*B, *tet*C, *tet*D, *qnr*D, *qnr*B, *qnr*A	Around Penglai	Maricultural environments	[35]
*sul*1, *sul*P, *tet*A, *tet*C, *qnr*D, *qnr*B	Around Lianyungang	Maricultural environments	[35]
*sul*1, *sul*3, *sul*P, *tet*B, *tet*C, *tet*D, *qnr*D, *qnr*B	Around Qidong	Maricultural environments	[35]
*sul*1, *sul*2, *sul*P, *tet*A, *tet*C, *tet*D, *qnr*S, *qnr*A	Around Xiangshan	Maricultural environments	[35]
*sul*1, *sul*2, *sul*3, *tet*A, *tet*C, *tet*D, *qnr*S, *qnr*A	Around Ningde	Maricultural environments	[35]
*sul*1, *sul*2, *sul*P, *tet*A, *tet*C, *tet*D, *qnr*D, *qnr*B, *qnr*A	Around Dongshan	Maricultural environments	[35]
*sul*1, *sul*3, *tet*B, *tet*C, *tet*D, *qnr*D, *qnr*S, *qnr*B	Around Zhanjiang	Maricultural environments	[35]
*sul*2, *sul*3, *tet*A, *tet*B, *tet*C, *tet*D, *qnr*D, *qnr*S, *qnr*B, *qnr*A	Around Lingshui	Maricultural environments	[35]
*tet*A, *tet*B	Around Meijijiao	Maricultural environments	[35]
